# Trichloroethylene: An Update on an Environmental Contaminant with Multiple Health Effects

**DOI:** 10.1146/annurev-pharmtox-022724-120525

**Published:** 2024-12-17

**Authors:** Lawrence H. Lash

**Affiliations:** Department of Pharmacology, Wayne State University School of Medicine, Detroit, Michigan, USA

**Keywords:** metabolism and pharmacokinetics, kidney, liver, neurotoxicity and Parkinson’s disease, immunotoxicity, reproductive and developmental toxicity

## Abstract

The halogenated solvent trichloroethylene (TCE) has had many uses in medicine, construction, consumer products, and the military. Many of these uses have been discontinued or restricted due to its toxicity, which affects multiple target organs and includes both acute, high-dose toxicity and chronic, low-dose toxicity that also encompass several cancers. US and international agencies have conducted risk and hazard assessments for TCE, with comprehensive publications coming out in the last 10–15 years. Accordingly, the focus of this article is to review recently published data since that time (i.e., 2014) that clarify unsettled questions or provide additional insights into the metabolism and mechanisms of toxicity of TCE in several target organs. Besides metabolism, the review focuses on the kidneys, liver, immune system, nervous system, cardiovascular and pulmonary systems, the search for biomarkers, and recent analyses of human cancer risk and incidence from TCE exposure.

## 1. INTRODUCTION

Trichloroethylene (TCE) is a nonflammable, chlorinated solvent that is a liquid at room temperature (boiling point of 89.6°C, melting point of −84.8°C) (1). TCE is considerably denser than water, with a density of 1.4642 at 20°C relative to that of water at 4°C (1). It has been produced commercially for approximately 100 years and has had many uses owing to its chemical properties and biological effects. As summarized in relatively recently published toxicological reviews by the US Environmental Protection Agency (EPA) (2), the International Agency for Research on Cancer (IARC) (3), and the National Toxicology Program (NTP) (4), TCE has been used as an anesthetic agent; a stain remover in dry cleaning; an ingredient in paints, adhesives, and cleaning fluids; an extraction agent for various food products; an intermediate in the synthesis of other chemicals, such as chlorofluorocarbons; and a metal-degreasing agent. Although many of the earlier exposures that were studied were occupational and at relatively high levels, TCE is also a significant contaminant of air, water, soil, food, and animal tissues and is a significant contaminant of EPA Superfund hazardous waste sites (5, 6). Accordingly, there has been a significant focus of public health risk assessment on TCE for many years, especially regarding chronic exposures that may result in various cancers.

Earlier assessments by the IARC of TCE as a human carcinogen (7) classified TCE as a Group 2A probable human carcinogen. This evaluation was based on sufficient evidence for carcinogenicity in animals but limited evidence in humans. Epidemiological data showed elevated risks of liver and biliary tract cancer and non-Hodgkin lymphoma. The reevaluation of TCE that occurred in 2012 and was published in 2014 (3) elevated TCE to a Group 1 known human carcinogen on the basis of strong epidemiological evidence of kidney cancer and strong mechanistic data from animal studies. Elevated risks of cancer in humans have been identified for the following sites: kidney, non-Hodgkin lymphoma, leukemia, liver and biliary tract, and cervix. Other cancers that have been identified in some studies, but not in others, include lung and bladder. Although cancer sites in other animal species and in humans differ somewhat, there is a general concordance between data in animals, such as rats and mice, and humans.

For noncancer effects of TCE, the major target organs include the kidneys, liver, immune system, male and female reproductive systems, cardiovascular system, neurological system, and pulmonary system. The strength of evidence for and sensitivity of each system or organ differ, with considerably more data being available for some than for others.

While the EPA risk evaluation was published in 2011 (2), the IARC evaluation of TCE was published in 2014 (3), and the NTP elevated TCE to a known human carcinogen in 2015 (4), interest has continued over the past decade in providing more accurate risk assessments for the various effects of TCE and better understanding of the mechanisms by which TCE affects different target organs both acutely and from chronic exposures. Moreover, a risk evaluation of TCE was performed in 2020 by the EPA to comply with the Toxic Substances Control Act (8). The EPA’s Chemical Assessment Advisory Committee concluded that TCE poses an unreasonable level of risk to human health.

Although the conclusions about the risks to human health posed by TCE have not substantially changed over the past decade, there has been a considerable increase in knowledge about TCE metabolism and the mechanism of action for several target organs. Accordingly, this review focuses on providing updates on TCE metabolism and pharmacokinetics and knowledge about the mechanisms of action in several target organs. The target organs or processes that are reviewed include the kidneys, liver, nervous system, immune system, cardiovascular system, pulmonary system, and reproductive and developmental toxicity. Additional topics that are reviewed include recent studies using proteomics, metabolomics, and transcriptomics to identify sensitive biomarkers of TCE effects and recent assessments of human cancer from TCE exposure.

Several thorough reviews published in the mid-2010s (9–12) have provided some recent updates and elaboration of data highlighted in the EPA (2) and IARC (3) toxicological assessments. This review endeavors to build on these publications, rather than repeat key issues, and highlights data and analyses published within the past 10 years. In doing so, the review assesses how complete the understanding is of the mechanism or mode of action in each target organ, assesses whether conclusions can be made about the sensitivity of each target organ relative to one another, and identifies knowledge gaps that need to be filled by additional research.

## 2. METABOLISM AND PHARMACOKINETICS

Metabolism of TCE to both stable metabolites and reactive intermediates has long been recognized as critical to the underlying chemistry and biochemistry of TCE-induced toxicity (10, 12, 13). Moreover, it has been established and accepted that virtually all of the adverse acute or chronic effects of TCE exposure are dependent on its metabolism. Accordingly, accurate quantification of metabolism is viewed as essential for at least two purposes. First, it provides a measure of dosimetry, potentially at the target cell level, to enable dose-response relationships to be established. Second, it provides more accurate information for physiologically based pharmacokinetic (PBPK) models. Such models are often created for species such as rats or mice and then used to extrapolate to humans. This is done in part because typically much more data are available from species other than humans and such data are needed to validate the models.

**Figure 1:**
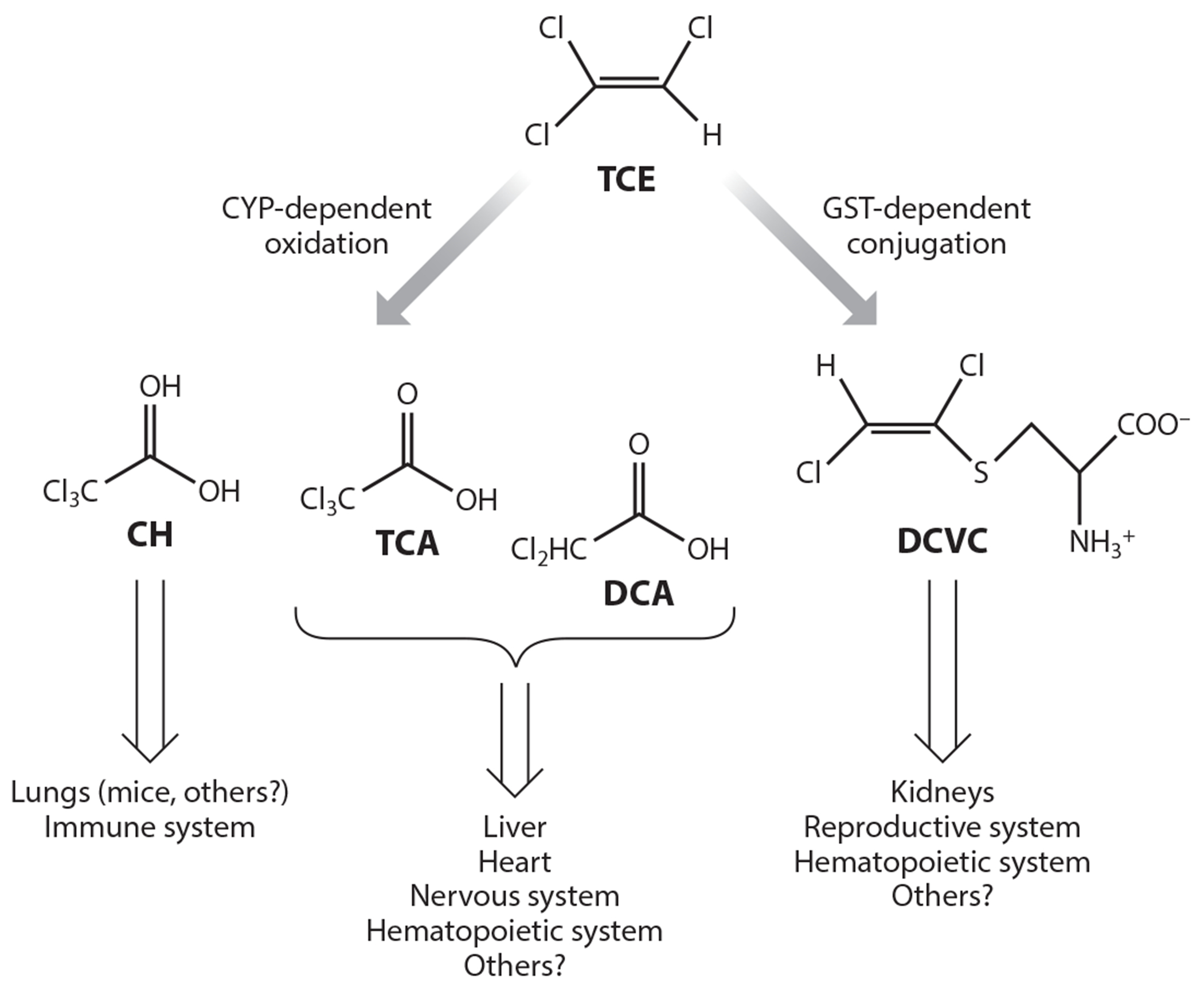
Relationship between trichloroethylene (TCE) metabolism and its target organ adverse effects. TCE can be metabolized by one of two pathways: an oxidative pathway initiated by cytochrome P450s (CYPs) or one initiated by glutathione (GSH) conjugation and catalyzed by glutathione *S*-transferases (GSTs). Major oxidative metabolites include chloral hydrate (CH), trichloroacetate (TCA), and dichloroacetate (DCA). The major, penultimate toxic metabolite generated from the conjugation pathway is the cysteine *S*-conjugate *S*-(1,2-dichlorovinyl)-l-cysteine (DCVC).

The link between TCE metabolism and its target organ-specific effects is illustrated in Figure 1. Metabolism is mediated by either of two pathways: one that begins with a cytochrome P450 (CYP)-dependent oxidation reaction or one that begins with a glutathione *S*-transferase (GST)-mediated conjugation reaction. While updates on research involving the various target organs of TCE are discussed in Section 3, this information is illustrated here to emphasize the importance of metabolism for the various modes of action of TCE.

A major issue regarding TCE metabolism that has existed for more than 25 years is the relative flux through the two main pathways, namely that initiated by CYPs and that initiated by GSTs, and what this implies for target organ toxicity of TCE. Due to the fundamentally different chemical nature of metabolites generated by the two pathways, analyses to quantify flux and assess toxicological importance must take this into account to avoid incorrect or incomplete conclusions. As described in earlier reviews (2, 3, 9, 10, 13), the fact that most of the CYP-derived metabolites are chemically stable whereas several of the GST-derived metabolites are reactive and chemically unstable resulted in dramatically higher recoveries of TCE metabolites such as trichloroacetate and trichloroethanol than of the mercapturate *N*-acetyl-*S*-(1,2-dichlorovinyl)-l-cysteine in urine in both rodents and humans exposed to TCE. This finding led to the conclusion that the GST-dependent pathway for TCE metabolism is quantitatively insignificant and that other mechanisms independent of GST-derived metabolism must exist to account for TCE nephrotoxicity and kidney cancer. In addition to this controversy and disagreement, there were disagreements in the literature during the 1990s and early 2000s about actual published rates of GSH conjugation of TCE. Such issues and the concept that flux of TCE through the GSH conjugation pathway is very small and toxicologically insignificant were discussed again in a 2018 paper (14).

Through development of highly sensitive and specific analytical methods and novel in vitro model systems, Rusyn and colleagues (15) validated the relatively high rates of glutathione (GSH) conjugation of TCE and those of its congener perchloroethylene. This controversy was reviewed in a commentary (16) that accompanied the publication by Rusyn and colleagues. An additional point that was emphasized in several papers (3, 9–13, 17) is that formation of TCE metabolites derived from the GSH conjugation pathway does not have to be comparable to that of metabolites derived from the CYP oxidation pathway for the former to be toxicologically significant. Again, this is readily explained by the knowledge that metabolites derived from GSH conjugation are highly reactive and chemically unstable. Because of these properties, a large proportion of these sulfur-derived metabolites are not recovered in urine as stable end products (i.e., mercapturates). Rather, a large portion of these metabolites interact with cellular macromolecules, including protein and DNA, produce toxicity, or decompose to other by-products. Thus, while urinary mercapturates may serve as an appropriate biomarker for exposure to TCE, they cannot be used to quantify metabolic flux through the GSH conjugation pathway. The critical impact of the analytical methodology developed by Rusyn and colleagues (15) is that more accurate metabolic flux data can be available to make better dosimetry predictions, potentially at the target tissue level, and more accurate PBPK models for human health risk assessment for TCE exposures.

Another major development regarding TCE metabolism during the past decade has been to use both PBPK models and different biological models to address the issues of species-, strain-, and tissue-dependent differences. For example, Chiu et al. (18) calibrated and extended existing murine models of TCE PBPK. Data on both oxidative and GSH conjugation metabolism of TCE in 16 inbred strains and 1 hybrid mouse strain were used. Use of genetically diverse mouse strains has become a valuable surrogate for estimating toxicokinetic variability in human populations. This study concluded that use of this approach, especially at the lower doses most relevant to environmental exposures, resulted in mouse population–derived variability for estimates of TCE metabolism that closely mimicked those obtained in human populations.

Tissue- and strain-dependent differences and other sources of variation in TCE metabolism have been recently studied using the more sensitive and specific methodology of Rusyn and colleagues (15) in experiments using different mouse strains (19) and using Cyp2e1 knockout and humanized transgenic mice (20). The results showed the importance but not exclusivity of Cyp2e1 in oxidative metabolism of TCE and that sex- and species-dependent differences in toxicity from TCE and the congener perchloroethylene can be explained by differences in metabolism.

Other studies by Rusyn and colleagues (21, 22) using their sensitive and specific methods for detection and quantitation of TCE metabolites showed that the distinct effects on liver and the kidneys are species, strain, and sex dependent and are correlated with differences in patterns or rates of metabolism. In livers (21), the key observations included interstrain variability in the amounts of TCE metabolites formed, the absence of induction of Cyp2e1 protein levels in liver, and modest induction of peroxisome proliferator marker genes, but significant increases in hepatocellular proliferation. In the kidneys (22), key observations included interstrain variability in levels of both CYP- and GSH-derived TCE metabolites; induction of peroxisome proliferator marker gene expression and increased cell proliferation as in the liver; and increases in the kidney biomarker of tubular injury and regeneration, kidney injury molecule-1 (Kim-1).

Commandeur and colleagues (23) demonstrated very large differences in the regioisomers of GSH and cysteine conjugates formed with TCE in rats and humans. Formation of either the 1,2-*cis*, 1,2-*trans*, or 2,2-isomers of dichlorovinylglutathione (DCVG) has a significant impact on toxic potency. These GSH conjugates are then converted to the corresponding cysteine conjugates, *S*-(1,2-*cis*-dichlorovinyl)-l-cysteine (DCVC), 1,2-*trans*-DCVC, and 2,2-DCVC. Commandeur and colleagues demonstrated that while 1,2-*cis*-DCVC and 1,2-*trans*-DCVC were readily converted to reactive species that formed cross-links with a model nucleophile, 2,2-DCVC could not alkylate the nucleophile and thus was deemed to be neither toxic nor mutagenic. These investigators also compared the abilities of rat and human liver cytosolic fractions to metabolize TCE to the various isomers of DCVG. Their key conclusions were that rat liver cytoplasm forms primarily 1,2-*cis*-DCVG, that human liver cytoplasm forms primarily the relatively inactive 2,2-DCVG, and that rates of DCVG formation were approximately 10-fold higher in rat liver cytoplasm than in human liver cytoplasm. Note, however, that measured rates of metabolite formation in rat and human livers contrast with those of Rusyn and colleagues (15), which agreed with the previously reported findings of Lash et al. (13).

In another recent study, Mortuza et al. (24) studied the pharmacokinetics of TCE in male Sprague-Dawley rats and compared them to that for its congener 1,1,1-trichloroethane (TRI). The authors found that while TRI exhibited linear toxicokinetics, TCE toxicokinetics was non-linear, with disproportionate increases in area under the curve and decreases in clearance with increasing dose. Total and first-pass hepatic elimination of TCE significantly exceeded those of TRI, whereas TRI exhibited a higher pulmonary elimination due to it having a higher air-to-blood partition coefficient than TCE. These interesting findings showed that despite TCE and TRI having several common physical and chemical properties, which resulted in similar absorption and systemic distribution, they displayed much different dosage and dose rate effects on their toxicokinetics.

Conclusions from the most recent studies of TCE metabolism and pharmacokinetics are that significant strain- and species-dependent differences exist in rates and patterns of TCE metabolism and that use of the most sensitive analytical methods has confirmed earlier data that emphasize the importance of the GSH conjugation pathway in the kidneys (13). However, additional work is needed to better define individual differences in humans and to relate these metabolic or pharmacokinetic differences to toxicity or risk of adverse effects.

## 3. RECENT DEVELOPMENTS IN UNDERSTANDING SENSITIVITY AND IMPORTANCE OF DIFFERENT TARGET ORGANS

This section describes recent work on several of the major target organs of TCE and its metabolites. For several of the target organs, a considerable amount of research had been previously done to establish various effects and mechanisms of action. The recent studies presented here generally represent new directions in research. A key question is how the newly described mechanisms relate to those previously established. In some cases, relationships between the various mechanisms can be inferred, whereas in other cases, additional research is needed to address this issue.

### 3.1. Kidney Injury: Mode of Action

While mechanistic studies of TCE and its GSH-derived metabolites in the kidneys that were conducted from the mid-1980s through approximately 2010 focused primarily on establishing the roles of processes such as mitochondrial dysfunction, oxidative stress, different intracellular enzymes in bioactivation and detoxification, and various plasma membrane transporters (2, 3, 9–13), most of the more recently published work on TCE-induced kidney injury has focused on processes related to the immune system and inflammation. While ultimately some of the same pathological processes as those described in the earlier work are activated and result in renal damage, these studies provide another layer of interacting systems that can be perturbed by TCE exposure.

A research group from Anhui Medical University in China has published an extensive series of studies investigating TCE-induced kidney injury in TCE-sensitized mice. These mice were developed to model the occurrence of occupational medicamentosa-like dermatitis due to TCE, which is a severe hypersensitivity reaction that occurs after TCE skin contact or inhalation and is associated with both hepatitis and kidney damage besides the characteristic effects on the skin.

In several studies, the group from Anhui Medical University demonstrated a role for several pathways or processes in various forms of immune-mediated renal injury, including plasma kallikrein-kinin (25); bradykinin (26); complement activation (27–30); high mobility group box-1 (HMGB1) (31); Wnt-5 and calcium signaling (32); cathepsin L and mammalian target of rapamycin (mTOR) signaling (33); a pathway involving sirtuin 1 (SIRT1), heat shock protein 70 (HSP70), and Toll-like receptor 4 (TLR4) (34); oxidative stress and NF-κB (26, 28, 35); endothelin-1 (30, 36); and necroptosis (37). The group also characterized the role of mitochondrial dysfunction, mitochondrial DNA damage, and communication with the immune system and the NLRP3 inflammasome in TCE-induced kidney damage (38–40). Thus, it is clear from these studies that several pathways become activated in TCE-sensitized mice, and these are then linked to renal injury by activating pathological processes such as oxidative stress, release of proinflammatory cytokines, and mitochondrial damage.

Although studies of the mechanism of TCE metabolite-induced kidney injury prior to 2014 showed in both in vitro and in vivo models direct effects on processes such as redox status and mitochondrial function, the more recent studies show that disturbances in these processes can also occur subsequent to immune system dysfunction. A critical issue that needs to be addressed is to determine the relative importance of immune system–mediated effects versus direct effects on redox state and mitochondrial function in producing renal cellular injury. It is likely that both mechanisms can occur simultaneously but that one may be more predominant than the other at either different doses/concentrations or different exposure times.

### 3.2. Liver Injury: Mode of Action

Previous research (2, 3, 9–12) on the effects of TCE and its metabolites on liver function demonstrated increased cell proliferation; nongenotoxic mechanisms of causing liver cancer, including changes in DNA methylation; and modest activation of peroxisome proliferator-activated receptor α (PPARα). Hepatic effects of TCE have been linked to CYP-dependent formation of metabolites, in particular trichloroacetate (TCA) and dichloroacetate (DCA). Recent studies of hepatic effects of TCE have focused on three main areas: (*a*) transcriptomics and associated regulatory mechanisms, (*b*) the role of the immune system, and (*c*) inflammation and the intestinal microbiome.

Chen and colleagues (41–44) studied epigenetic changes in the livers of TCE-exposed rats and mice, including alterations in DNA methylation, gene expression, and expression of long intergenic noncoding RNAs (lincRNAs). The studies showing changes in DNA methylation extended earlier work and showed that expression of several genes in mouse liver associated with DNA methylation were dysregulated (41). Studies of rats exposed to TCE found that 806 genes were hypermethylated but that 186 genes were hypomethylated (43). Moreover, these altered methylated genes were associated with several pathways representing important processes that can lead to liver toxicity and liver cancer, including endocytosis, mitogen-activated protein kinase (MAPK), and cyclic AMP signaling pathways. Transcriptional profiling of mouse liver DNA after TCE exposure showed alterations in key signaling pathways related to cell proliferation, PPARα, apoptosis, homologous recombination, and DNA methylation (42, 45). One of these studies (45) also showed a prominent effect on expression of genes related to fatty acid metabolism and differences in response and dose dependence in different mouse strains. RNA microarrays showed that more than 100 lincRNAs were dysregulated, and the studies identified specific lincRNAs that are associated with several cancer-related signaling pathways, such as PPARs and cell cycle regulation (44).

As with studies of kidney toxicity associated with TCE exposure discussed above, by far the largest number of recent studies of liver toxicity associated with TCE exposure have focused on inflammation and the immune system (46–55). Luyendyk and colleagues (46, 47) used a mouse strain that spontaneously develops autoimmune cholangitis and is used as a model of primary biliary cholangitis to examine the effects of TCE exposure on liver function. Whereas the authors reported that TCE was profibrogenic in one study (46), TCE significantly reduced autoimmune liver injury in mice in another study (47). The authors suggested that the precise effect of environmental chemicals on autoimmunity depends on the experimental model and is obviously a complex process.

Blossom and colleagues (48) focused on autoimmune hepatitis-like disease in male and female mice, using a strain that is prone to development of autoimmunity. Although exposure conditions were such that none of the mice actually developed pathological or serological signs of the autoimmune disease, TCE-exposed female mice were more likely than TCE-exposed males to exhibit higher levels of liver biomarkers associated with regeneration, repair, and proliferation. These results are consistent with the higher incidence of autoimmune diseases in females and illustrate the important role of the immune system in responses of the liver to environmental toxicants such as TCE.

The key role of the kallikrein-kinin system and Kupffer cells and other macrophages in the immune system and TCE-induced liver toxicity was emphasized in another series of studies (49–55). The kallikrein-kinin system and bradykinin were also involved in TCE-induced kidney injury (25, 26). In the liver, the data clearly show a promotion of release of proinflammatory cytokines by TCE exposure, including tumor necrosis factor-α (TNF-α)/TNF receptor 1 (TNFR1) and various interleukins, and alterations in MAPK pathways.

As with the recent studies of kidney toxicity described above, a key issue regarding the liver for additional research involves the relationship between the various proinflammatory effects and immunotoxicity with previous data on liver toxicity from TCE exposure. Like the situation with the kidneys, it is likely that multiple mechanisms occur simultaneously in the liver and that the relative importance of each differs with varying doses/concentrations and exposure times.

### 3.3. Neurotoxicity and Parkinson’s Disease

Although the potential neurotoxicity of TCE has long been recognized, mechanistic evidence for adverse neurological effects at environmentally relevant doses or concentrations has been lacking (2, 3). Rather, most of the evidence for neurotoxicity has been at very high levels of exposure. Nonetheless, use of TCE as an anesthetic agent was prohibited in the United States only in 1977 (7).

Several recent studies have examined rather subtle, adverse effects of TCE on the nervous system. For example, in a study using MRL^+/+^ mice (autoimmune-prone strain), Meadows et al. (56) hypothesized that neuroinflammatory markers such as IL-6 and IL-10 would be altered by TCE exposure. What they found was that developmental exposure to TCE resulted in an initial compensatory response to early neuroinflammation. Other studies have demonstrated toxicity of TCE in rat neuroprogenitor cells (57), neuroinflammation and oxidative stress in mice when exposed prenatally to TCE (58), and dopaminergic neurodegeneration in rats and mice after low-dose inhalation of TCE (59). Epigenetic changes in rat cerebellar DNA have also been noted after chronic, developmental exposure to low levels of TCE (60). A final example that highlighted more of a neurobehavioral effect of TCE exposure was a recent case study reporting the development of unusual anxiety in an otherwise healthy 24-year-old male that was associated with occupational exposure to TCE (61).

Considerable attention has been given in recent years to the potential association between environmental exposure to TCE and the development of Parkinson’s disease (PD) (62–65). The weight of evidence has progressed to where many have come to consider PD to be largely an environmental disease (65). As noted above and described by Adamson et al. (59) and Liu et al. (66), evidence has been obtained that TCE exposure can result in degeneration of dopaminergic neurons. This occurs in the nigrostriatum and was associated with motor and gait impairments. The authors also observed accumulation of pSer-α-synuclein in dopaminergic neurons and microglial activation in the substantia nigra, suggesting that TCE inhalation in rats or mice causes potential dopaminergic degeneration and can mimic some of the neuropathology that has become associated with PD.

Additional mechanistic studies have also demonstrated dopaminergic degeneration associated with TCE exposure and suggested that the underlying mechanism may involve induction of leucine-rich repeat kinase 2 (LRRK2) kinase activity by TCE (67). Mutations in *LRRK2* are the most common genetic cause of familial and sporadic PD, and elevated activities of LRRK2 kinase are believed to contribute to dopaminergic neurodegeneration. Finally, alterations in the intestinal microbiome appear to be a common feature of patients with PD. Studies by Ilieva et al. (68) suggest that TCE exposure directly causes changes in the distribution of bacterial species in the intestinal microbiome, leading to increased risk of chronic diseases, including PD.

Hence, there seem to be multiple, potential mechanisms by which TCE can cause neurodegeneration. What is uncertain, however, is the role of each mechanism and the tissue dosimetry that results in sufficient exposures to TCE, especially in the environment, that can cause these neurological effects.

### 3.4. Hypersensitivity Syndrome, Immunotoxicity, and Inflammation

A target organ that has been studied extensively in the past decade is the immune system (69). Besides the other target organ toxicities discussed thus far, occupational exposure to TCE can also cause a systemic skin disorder with hepatitis known as TCE hypersensitivity syndrome (THS) (70, 71). Determinants of TCE hypersensitivity are not clearly known. However, several observations have been made that suggest some underlying mechanisms. For example, worsened THS has been associated with increased urinary TCA (72), suggesting a correlation with oxidative metabolism. Increased serum levels of antibodies for CYP2E1 IgG (73) and genetic polymorphisms in the HLA (74, 75) are associated with altered severity of THS.

In a recent review/commentary on the epigenetic basis for immunotoxicity and autoimmune disease, Blossom & Gilbert (76) proposed the concept that there is significant evidence that environmental exposures play a large role in development of immune system dysfunction. The key rationale they cite is that the concordance rate for development of autoimmune disease in identical twins is only approximately 50%. Moreover, they propose that exposure to immunotoxic chemicals early in life, when the immune system is still developing and likely highly sensitive to such agents, may be responsible for much of the adult-onset immunotoxicity and autoimmune disease. Blossom & Gilbert go on to discuss evidence that environmental pollutants such as bisphenol-A, mercury, 2,3,7,8-tetrachlorodibenzo-*p*-dioxin, and TCE all have in common the ability to produce epigenetic changes by altering patterns and amounts of DNA methylation, thereby leading to altered function of the immune system. Gilbert and colleagues (77–79) published a series of studies showing that developmental exposure to TCE leads to alterations in DNA methylation, including in CD4^+^ T cells. Phillips et al. (80) conducted an epigenome-wide association study of TCE-exposed workers and found variability in the blood DNA methylation profile, such that human exposure to TCE was associated with epigenetic alterations in genes involved in cell matrix adhesions and interferon subtype expression, which are important in the development of autoimmune diseases, and in genes related to cancer development.

Other studies have described the role of nitrosative and oxidative processes in TCE-mediated autoimmunity (81–84), including activation of signaling pathways involving TLR4, Nrf2, and the NLRP3 inflammasome. As with other target organs, TCE-induced immunotoxicity is dependent on its metabolism. Multiple studies suggest that TCE-induced immunotoxicity is dependent on its oxidative metabolism (85–87). In one study (85), both TCA and DCA produced the same effects as the parent compound TCE on inflammatory cytokines and other T cell activation markers. Another study (86), however, provided evidence that THS was mediated by the TCE metabolite chloral hydrate. Further support for the importance of oxidative metabolism of TCE in immunotoxicity is the demonstration of diminished susceptibility of *Cyp2e1* knockout mice to TCE-induced autoimmunity (87). One recent study by Harris et al. (88) showed that the cysteine conjugate of TCE, namely DCVC, suppresses inflammatory responses of THP-1 human macrophages, suggesting that TCE immunosuppression can also occur through the GSH conjugation pathway. Additional processes that are believed to play a role in TCE-induced autoimmunity include differential expression of microRNAs (miRNAs) (89) and changes in the intestinal microbiome (90, 91).

Thus, while the existence of immune system dysfunction as an adverse response to TCE exposure has been well established, it is not clear which metabolite is responsible for the observed effects, what the key mechanisms of immunotoxicity are, and what the risk relative to that for other target organ adverse effects is.

### 3.5. Cardiotoxicity and Developmental Heart Defects

The heart and cardiovascular system are not targets for TCE-induced acute toxicity in the same manner that the liver, kidneys, and immune system are targets. Rather, TCE exposures have been associated with congenital heart defects or developmental cardiotoxicity (92, 93). TCE also disrupts cardiogenesis in human embryonic stem cells (94). While the mechanism by which TCE causes developmental cardiotoxicity has not been clearly elucidated, a few studies have recently demonstrated a role for the transcription factor hepatocyte nuclear factor 4-alpha (HNF4α) (95, 96), the arylhydrocarbon receptor (97), and a specific miRNA (98). Serum levels of methylated arginine have been recently proposed as a biomarker of TCE cardiovascular risk (99).

The considerable uncertainty about results from different studies of developmental cardiotoxicity caused by TCE exposures that was described in the EPA risk assessment document in 2011 (2) still exists. This is largely because limitations of the various studies have been deemed to be unresolvable (92). Clearly, additional studies are needed to resolve the inconsistencies in the data and determine the true risk.

### 3.6. Pulmonary Toxicity

The lungs and pulmonary system are not considered a major target organ of TCE in humans (2, 3). Of the various animal species used to study TCE-induced toxicity, lung damage is observed only in mice, where the unique, species-specific accumulation of chloral hydrate is believed to result in acute injury to Clara type 2 pulmonary epithelial cells. Due to this mouse-specific metabolic pattern and the differences between mice and humans regarding pulmonary epithelial cell types, this effect of the TCE metabolite chloral hydrate on the lungs is considered to be a mouse-specific effect and therefore not relevant to humans.

Nonetheless, there have been some reports of respiratory effects associated with TCE exposure, although results are conflicting. As summarized in a recent review article (100) and in the most recent IARC evaluation of TCE (3), there is limited evidence of an increased risk of lung cancer associated with TCE exposure based on animal and human data. Some of the concerns with these data include the existence of mixed exposures to multiple chlorinated solvents, thereby confounding the results. However, there are limited data available to support an association between TCE exposure and other types of respiratory tract disorders, such as asthma, chronic bronchitis, or rhinitis (100). Again, there are some conflicting results, but the most consistent data are related to TCE-induced autoimmune and vascular diseases that also affect pulmonary function.

A recent case study (101, p. e907) reports an incident of combined pulmonary fibrosis and emphysema in an aircraft maintenance worker who had what was described as “prolonged and intense exposure” to TCE.

### 3.7. Reproductive and Developmental Toxicity

TCE, through its metabolites, causes reproductive or developmental toxicity. Studies of rodents (102) and the zebrafish model (103) have shown that exposure to TCE results in adverse pregnancy results and responses, such as decreased fetal body weight. The mechanisms by which this toxic response occur, however, are less clear. Some studies have found either TCE or some of its metabolites to be endocrine disruptors (104). Immunotoxicity has also been proposed as the basis for developmental toxicity of TCE (105). Data have clearly shown that the placenta is a target organ of TCE (106, 107). While a few studies have shown some effects associated with exposures to high doses of CYP-derived metabolites of TCE (e.g., 104), most of the data have focused on DCVC as the penultimate toxic agent in TCE-induced reproductive and developmental toxicity. In one study (108), the ability of DCVC to stimulate release of TNF-α in an extraplacental membrane preparation was directly compared with that of TCA; only the former stimulated production of this cytokine.

DCVC produces several adverse effects in human trophoblast cell lines (109–112). These effects have included elevated levels of reactive oxygen species, increased production of inflammatory cytokines, alterations in cellular energy metabolism and redox regulation, and mitochondrial dysfunction. An interesting observation that has important implications for development and reproductive success is that BeWo cells, which are a human placental trophoblast model, can be induced to undergo the process of syncytialization by treatment with forskolin. Syncytialization is a process in the placenta whereby cytotrophoblasts differentiate to form syncytiotrophoblasts and involves multinucleation and cell fusion steps. This process is critical for a healthy pregnancy, as it helps form the fetal–placental interface. Notably, DCVC-induced changes in cellular function and toxicity were more severe when BeWo cells either were undergoing or had completed syncytialization (111, 112). Some of the same metabolic and energy metabolism changes were also observed in the amniotic fluid of rats treated with DCVC (111), validating the in vitro results.

Although some TCE metabolism data for reproductive organs are available, it remains unclear whether sufficient levels of key metabolites that promote toxicity are produced, under what conditions they are produced, and whether it is CYP-derived or GST-derived metabolites that are most important in producing adverse outcomes.

## 4. BIOMARKERS: PROTEOMICS, METABOLOMICS, AND TRANSCRIPTOMICS

The search for sensitive and specific biomarkers of TCE adverse effects, especially prior to the occurrence of significant and irreversible injury, has been a longstanding goal. Interest in identifying such biomarkers for the kidneys has been especially prominent for multiple reasons (113). First, the occurrence of acute kidney injury and incidence of chronic kidney disease have remained stubbornly high for several decades despite advances in health care. Second, the kidneys are well known to have the capacity for repair and regeneration as long as the insult is not too severe. While several biomarkers for the kidneys have been discovered and validated over the past 10–20 years, none of these are linked to the mechanism of action of kidney injury–inducing chemicals and they are nonspecific.

Recognizing that the mitochondria of the renal proximal tubular cell are prominent, sensitive, and early targets for many nephrotoxicants, including the TCE metabolite DCVC, researchers have hypothesized that this organelle may be a source of new biomarkers (114, 115). Walker et al. (116) have also used high-resolution metabolomics to identify changes in metabolic pathways from urine samples of workers exposed to TCE. Even though exposures were below recommended limits, metabolic changes, including those in metabolism of purines, bile acids, and sulfur amino acids, were consistently observed.

Proteins that are secreted from cells are also a potential source of biomarkers. Serum markers for TCE-induced immune system dysfunction and inflammation, including occupational medicamentosa-like dermatitis, have been observed in workers occupationally exposed to TCE (117–119). Besides proteins, miRNAs and transcriptomics have been used to try to identify new markers of TCE exposure (120, 121). This area remains an emerging field.

## 5. HUMAN CANCER EVIDENCE

Several recent reviews and epidemiological analyses have been conducted in the decade since the release of the more recent IARC monograph on TCE (3). Most of these have focused on kidney cancer due to either occupational exposures (122–124) or a general population-based analysis that includes exposure via groundwater contamination (125, 126). There is consensus that both kidney cancer and non-Hodgkin lymphoma in humans were the driving forces for evaluating TCE as a known human carcinogen. Another relatively recent study focused on blood DNA markers related to immune system cancers (80). While these more recent studies have not changed the primary target organs or relative sensitivities of the different target organs identified in the major risk or hazard assessment documents (2–4), they have provided additional data that support those conclusions.

## 6. CONCLUSIONS

This review provides an update on research developments concerning the environmental pollutant and human carcinogen TCE and highlights areas that are still controversial or evolving. While this is a rather daunting task, considering the large number of papers published on TCE over the years, the approach taken was to use the risk assessment publications by the EPA (2), IARC (3), and NTP (4) as the starting point. Studies published in the past decade (i.e., 2014 and later) were collected to summarize new information and new approaches that have helped to resolve longstanding controversies or inconsistencies in the database. Thus, while an enormous database of metabolism, toxicity, carcinogenicity, and other mechanistic studies already existed prior to the risk assessment publications from about a decade ago, it should be clear from this review that a significant amount of new research conducted in recent years has achieved several goals.

First, controversies and discrepancies in the literature about quantitative data on TCE metabolism have been recently clarified, thereby providing more accurate information and values for dosimetry and pharmacokinetics. New information has included a better understanding of species-, strain-, and sex-dependent differences in metabolism. This information can improve risk assessments and predictions of interindividual differences that alter risk. It remains to be clarified, however, how these data translate to differences that might exist in human populations due to factors such as diet, age, genetic polymorphisms in key enzymes, and preexisting diseases or chronic conditions.

Second, recent work has provided new mechanistic information about the various target organs affected by TCE exposure. Table 1 provides a broad summary of the major target organs of TCE and whether toxicity data for acute or chronic exposures are available from humans and/or animals. These target organs include the kidneys, liver, immune system, cardiovascular system, reproductive systems, and development. Additionally, recent novel studies that identify biomarkers for TCE effects were briefly described. Altered homeostasis in the intestinal microbiome has also been implicated in mediating some of the effects of TCE on target organs. The potential influence of the microbiome is an emerging area in many aspects of modern biology and requires continued efforts to understand its role in regulating several key cellular processes and functions.

One of the key messages from reviewing these recent, target organ studies is the prominent role of the immune system as it affects other organ systems. Thus, in addition to a large number of investigations into TCE-induced autoimmunity and TCE-induced dysregulation of the immune system, numerous studies have demonstrated a critical role for TCE-mediated changes in immune system function and inflammation leading to renal or hepatic toxicity. Some of these immune system changes also lead to activation of certain pathological processes such as release of reactive oxygen species or inflammatory cytokines. While previous studies have identified changes in redox status or processes such as mitochondrial function, the relative roles remain unclear of the direct effects of TCE or its metabolites on these processes or the effects on these processes resulting from the immune system or inflammation.

Although neurotoxicity of TCE has been known for some time, recent studies have demonstrated underlying mechanisms by which TCE exposure results in neurodegeneration, especially involving dopaminergic neurons. Accordingly, the concept has been promoted that although there are genetic disease determinants, PD may also be the result of environmental exposures. TCE exposure has been implicated as one cause of PD.

Cardiovascular and pulmonary toxicity of TCE is in a category different from that of the other target organs. For the cardiovascular system, there is little information from humans and few data from human systems. For the animal data, however, the clearest and most consistent findings relate to developmental effects. For pulmonary toxicity, there is some weak evidence of an elevated risk of lung cancer in TCE-exposed humans but very little data supporting acute effects. The data that do support acute pulmonary effects are related primarily to immune system or inflammation-mediated responses. The clearest data on acute effects of TCE exposure on pulmonary function come from studies of mice and are considered not relevant to humans due to unique metabolic and cellular features of the mouse lung.

Recent studies have used approaches such as proteomics, metabolomics, and transcriptomics to identify novel biomarkers of TCE exposure in both clinical samples and experimental systems. These studies are at their early stages and promise to provide both mechanistic insight and new opportunities for toxicity treatment or prevention.

Finally, only a few studies of human cancers associated with TCE exposures have been published in the decade since publication of the IARC TCE monograph (3). A few epidemiological studies describing kidney cancer risk in humans and one study focusing on immune system–related cancers in the blood in humans were reviewed. These are significant studies because kidney cancer and blood cancers such as non-Hodgkin lymphoma in humans were the driving forces for the elevation of TCE to a Group 1 known human carcinogen.

Hence, recent research has emphasized the importance of effects on the immune system and inflammation in the mode of action of TCE. These effects include both disturbances in the immune system itself and immune-mediated effects on other target organs, especially the kidneys and liver. It remains unclear whether these immune system–mediated effects are driving kidney and liver toxicity or whether direct effects of TCE metabolites on biochemical processes such as redox state and mitochondrial function are the drivers of target organ dysfunction. The development of novel, highly sensitive analytical methods for quantifying metabolism holds the promise of more accurate dosimetry at the target tissue or even cellular level.

## Figures and Tables

**Table 1:** Summary of the target organs of trichloroethylene

Target organ	Acute injury^a^	Chronic injury/cancer^a^
Kidneys	H, A; strong consensus	H, A; strong data for cancer in humans
Liver	H, A; consensus but most prominently in male rat	H, A; evidence modest or inconsistent in humans
Nervous system	H, A; high-dose effect	H, A; neurodegenerative disease
Immune system/hematopoietic	H, A; strong consensus	H, A; non-Hodgkin lymphoma, leukemia
Cardiovascular	No data	A; developmental
Lungs	H (weak), A; mostly observed in mice	No data
Reproductive and developmental	H, A; human data in vitro	A; developmental

a Comments indicate whether support comes from human (H), animals (A), or both and the consensus or clarity of the data.

## SUMMARY POINTS

1. This review provides an update of research on trichloroethylene (TCE) metabolism and toxicity over the past decade since the publication of risk assessments by US federal and international organizations.
2. Sensitive analytical methods and novel in vitro models have been used to resolve controversies regarding rates of glutathione-dependent metabolism of TCE and its congener perchloroethylene. These more accurate measurements of metabolic flux can be used to improve the predictivity of physiologically based pharmacokinetic models and accuracy of target tissue dosimetry.
3. Recent studies of TCE-induced kidney and liver injury have focused primarily on immune system– and inflammation-mediated mechanisms. Some of these mechanisms led to previously identified pathological responses, including oxidative stress, release of proinflammatory cytokines, and mitochondrial dysfunction.
4. Recent studies have demonstrated underlying mechanisms by which TCE exposure can cause neurodegeneration, especially involving dopaminergic neurons. Epidemiological and experimental data support a role for environmental TCE exposure as a cause of Parkinson’s disease in humans.
5. Numerous studies have demonstrated a critical role for TCE-mediated changes in immune system function and autoimmune diseases.
6. TCE exposure causes developmental cardiotoxicity, although the underlying mechanism remains unclear.
7. While the lungs are not considered a primary target organ of TCE in humans, there is weak epidemiological evidence for an increased risk of lung cancer from TCE exposure. There is some evidence of pulmonary dysfunction mediated by TCE-induced changes in the immune system.
8. There has recently been an increased focus on the search for sensitive biomarkers of TCE effects using proteomics, metabolomics, and transcriptomics.

## FUTURE ISSUES

1. Although the kidneys are one of the most studied target organs of TCE, questions remain about the importance of specific reactive metabolites, identifying the key bioactivation enzymes for TCE and its downstream metabolites, and tissue dosimetry related to organspecific effects.
2. The importance of immune system–mediated effects on redox status and mitochondrial function in the kidneys and liver compared with direct effects of TCE metabolites on these processes requires additional research.
3. Species-, strain-, and sex-dependent differences in metabolism have provided insights into variations in human responses. This information can improve risk assessments and improve predictions of interindividual differences that alter risk.
4. For the kidneys and other target organs that exhibit cancer as a response to chronic exposure, a key issue that requires more research concerns the relative roles of genotoxic versus epigenetic mechanisms.
5. Uncertainties remain about the liver as a target organ of TCE. Additional studies are needed to clarify how some of the mechanisms identified in studies of rodents may apply to humans.
6. The perturbations in the immune system caused by TCE exposure and the relationship between genetics and TCE exposure in autoimmune diseases continue to be an area of intense interest. Continued studies are needed to better understand these relationships and to identify specific targets for therapeutic intervention.
7. Cardiovascular defects in development that appear in some studies to be associated with TCE exposure require additional work to delineate mechanisms and identify targets for therapeutic intervention.
8. The search for sensitive biomarkers of TCE effects using various ’omics methodologies has become an active area of research with the goal of identifying specific markers that can readily translate to humans and be used to detect low-dose, environmental exposures before significant target organ damage occurs.

## Abbreviations

Biomarker: typically a metabolite or protein that is released from tissues in association with exposure to a chemical agent and subsequent effects on target organs
Physiologically based pharmacokinetic (PBPK) model: a mathematical model of how a chemical enters the body, translocates between tissues, is metabolized, and is excreted
Cytochrome P450 (CYP): family of heme-containing enzymes that catalyzes a diverse array of reactions, including oxidation of TCE
Glutathione *S*-transferase (GST): family of enzymes found in most tissues but especially the liver that catalyze the conjugation of electrophilic substrates with glutathione
Target organ toxicity: specific adverse effects from a chemical exposure on specific organs or organ systems
Mercapturate: an *N*-acetyl-l-cysteine conjugate that is the terminal metabolite derived from glutathione conjugation and is often recovered in urine
*S*-(1,2-dichlorovinyl)-l-cysteine (DCVC): cysteine conjugate derived from conjugation of TCE with glutathione; it is the penultimate metabolite associated with kidney and reproductive toxicity
TCE-sensitized mice: mouse strains that are genetically programmed to develop autoimmune diseases after exposure to TCE
Peroxisome proliferator-activated receptor α (PPARα): a ligand-activated transcription factor that regulates the expression of genes involved in fatty acid and energy metabolism
Epigenetic changes: changes in gene expression resulting from changes in DNA besides the genetic code; involve heritable traits that occur in the absence of changes in gene sequence; hypo- and hypermethylation are common forms
TCE hypersensitivity syndrome (THS): occupational exposure to TCE by skin contact or inhalation can cause a systemic skin disorder with hepatitis and kidney damage
BeWo cells: a human placental trophoblast cell line that can be induced to undergo the process of syncytialization by treatment with forskolin
Syncytialization: a process in the placenta whereby cytotrophoblasts differentiate to form syncytiotrophoblasts and involves multinucleation and cell fusion steps
